# Radiographic features in investigated for *Pneumocystis jirovecii* pneumonia: a nested case-control study

**DOI:** 10.1186/s12879-020-05217-x

**Published:** 2020-07-10

**Authors:** Jimmy M. Hsu, Aaron Hass, Marc-Alexandre Gingras, Jaron Chong, Cecilia Costiniuk, Nicole Ezer, Richard S. Fraser, Emily G. McDonald, Todd C. Lee

**Affiliations:** 1 Faculty of Medicine, McGill University, Montreal, Canada; 2 Department of Medicine, McGill University, Montreal, Canada; 3 Department of Radiology, McGill University, Montreal, Canada; 4 Clinical Practice Assessment Unit, McGill University Health Centre, Montreal, Canada; 5 McGill Interdisciplinary Initiative in Infection and Immunity, Montreal, Canada; 6 Department of Pathology, McGill University, Montreal, Canada; 7 McGill University Health Centre / Royal Victoria Hospital, McGill University, 1001 Décarie Blvd, E5.1820, Montreal, QC H4A 3J1 Canada

**Keywords:** *Pneumocysitis jirovecii*, PCP, PJP, Pneumonia, Computed tomography, Diagnosis

## Abstract

**Background:**

*Pneumocystis jirovecii* pneumonia (PJP) can be challenging to diagnose, often requiring bronchoscopy. Since most patients suspected of PJP undergo imaging, we hypothesized that the findings of these studies could help estimate the probability of disease prior to invasive testing.

**Methods:**

We created a cohort of patients who underwent bronchoscopy specifically to diagnose PJP and conducted a nested case-control study to compare the radiographic features between patients with (*n* = 72) and without (*n* = 288) pathologically proven PJP. We used multivariable logistic regression to identify radiographic features independently associated with PJP.

**Results:**

Chest x-ray findings poorly predicted the diagnosis of PJP. However, multivariable analysis of CT scan findings found that “increased interstitial markings” (OR 4.3; 95%CI 2.2–8.2), “ground glass opacities” (OR 3.3; 95%CI 1.2–9.1) and the radiologist’s impression of PJP being “possible” (OR 2.0; 95%CI 1.0–4.1) or “likely” (OR 9.3; 95%CI 3.4–25.3) were independently associated with the final diagnosis (c-statistic 0.75).

**Conclusions:**

Where there is clinical suspicion of PJP, the use of CT scan can help determine the probability of PJP. Identifying patients at low risk of PJP may enable better use of non-invasive testing to avoid bronchoscopy while higher probability patients could be prioritized.

## Background

*Pneumocystis jirovecii* is a fungal organism that is a frequent cause of opportunistic pneumonia (PJP) in immunocompromised patients with and without HIV [1, 2]. Making the diagnosis can be challenging, requiring a respiratory specimen from induced sputum or bronchoalveolar lavage (BAL) be obtained for direct immunofluorescent or cytologic staining [1]. Unfortunately, induced sputum is not always possible, and bronchoscopy carries a risk of complications. Nonetheless, establishing a definitive diagnosis of PJP remains important because treatment based on clinical presentation alone is associated with a worse prognosis [3]. Consequently, better use of empiric therapy and improved case selection for bronchoscopy can both improve the quality of care. Such an improvement begins with a better estimate of the likelihood of PJP compared to other diagnoses.

Fortunately, most patients who are suspected of PJP will have imaging of the lungs. Previous studies have suggested that computed tomography (CT) of the chest is more sensitive than chest x-ray (CXR) for the detection of PJP and furthermore, that the most common CT finding is the presence of ground-glass opacities [1, 2, 4–6]. We sought to determine if the differences in imaging findings between those with and without pathologically confirmed PJP could help better estimate the probability of PJP.

## Methods

The McGill University Health Centre (Montréal, Canada) is a 770-bed tertiary care hospital which serves as a referral center for: HIV/AIDS; rheumatologic disease; solid organ transplantation (liver, kidney, pancreas, and heart); and autologous and allogeneic stem cell transplantation. We identified all patients who had a BAL and/or transbronchial biopsy which was tested for pneumocystis using Calcofluor staining between January 2015 and January 2018. We then manually reviewed each patient’s bronchoscopy documentation and removed those where diagnosing PJP was not the reason for the bronchoscopy. From the remaining cohort, we selected all cases with pathologically confirmed PJP. For each case we randomly selected 4 controls who underwent bronchoscopy during the same timeframe to investigate for PJP but who tested negative. Controls were not matched on any other variables. We extracted readily available demographic information. We then extracted the full text radiology reports for the X-Rays and CT scans performed closest (but prior) to bronchoscopy and coded them based on categories of pertinent findings. The radiologist’s final impression was classified as: PJP not mentioned or unlikely, PJP possible, or PJP likely.

Univariable analyses were performed for both CXR and CT scan reports to evaluate for associations between the radiographic features and the final diagnosis of PJP. Multivariable analysis used logistic regression and a stepwise backwards elimination method beginning with features identified in the univariate analysis as potentially associated (*p* ≤ 0.2) while attempting to optimize the Akaike Information Criterion [7]. Independently associated variables (*p* < .05) were retained in the final models. For CT scan, we created a separate model without the radiologist’s impression to allow for an assessment of the radiographic features alone. Internal validation of each final multivariable model was performed using bootstrapping with 200 repetitions and the estimate of model optimism was used to generate a bootstrap corrected c-statistic [8]. A nomogram to estimate the probability of PJP based on CT scan features was created using the STATA program “nomolog”. All analyses used STATA version 15 (StataCorp LP, USA).

## Results

860 patients underwent testing for pneumocystis of whom 626 (72.8%) were deliberately investigated for PJP. From the latter group, 72 unique cases (11.5% positivity) were paired with 288 randomly chosen controls without PJP. The median age of cases and controls was 58 (IQR: 49.75–67.5) and 62 (51–69) respectively. 62.5% of cases and 61.2% of controls were male. HIV infection was present in 29.1% of cases and 8.6% of controls (*p* < 0.001).

The radiographic features of the CXRs (*n* = 69 cases/270 controls) and CT scans (*n* = 64 cases/231 controls) are shown in Table 1. Following multivariable analysis of CXR features, only the radiologist’s impression that PJP was possible or likely (OR 4.5; 95%CI 1.8–10.9) and the presence of “increased interstitial markings” (OR 2.9; 95%CI 1.6–5.1) remained independently associated with the diagnosis of PJP (adjusted c-statistic 0.64). Multivariable analysis of CT scan features demonstrated that “increased interstitial markings” (OR 4.3; 95%CI 2.2–8.2), “ground glass opacities” (OR 3.3; 95%CI 1.2–9.1) and the radiologist’s impression of PJP being “possible” (OR 2.0; 95%CI 1.0–4.1) or “likely” (OR 9.3; 95%CI 3.4–25.3) were independently associated with the diagnosis (adjusted c-statistic 0.75). In the model for CT scan without the radiologist’s impression, “increased interstitial markings” (OR 5.0; 95%CI 2.6–9.7) and “ground glass opacities” (OR 4.2; 95%CI 1.5–11.5) were positively associated with the diagnosis, whereas the presence of pleural effusion(s) (OR 0.44; 95%CI 0.22–0.91) and “nodular findings” (OR 0.41; 95%CI 0.21–0.81) were negatively associated (adjusted c-statistic 0.74). Nomograms to estimate the post-CT probability of PJP are presented in Table 2 and assume a prevalence of PJP similar to ours.

**Table 1:** Radiology findings and interpretation of cases and controls

	Univariable			Multivariable
X-ray Finding (%)	Cases (N = 69)	Controls (***N*** = 270)	***P***-Value	Odds Ratio (95% CI)
Increased interstitial markings	33 (47.8)	50 (18.5)	0.0001	2.9 (1.6–5.1)
Pleural Effusion	13 (18.8)	72 (26.7)	0.18	–
Airspace Disease	12 (17.4)	70 (25.9)	0.14	–
Atelectasis	10 (14.3)	44 (16.3)	0.68	–
Consolidation	8 (11.6)	43 (15.9)	0.37	–
Ground glass infiltrate	8 (11.6)	12 (4.4)	0.02	–
Reticulo-nodular opacities	7 (10.1)	30 (11.1)	0.82	–
Pulmonary edema	6 (8.7)	37 (13.7)	0.26	–
Normal	1 (1.4)	14 (5.2)	0.18	–
Radiologist PCP Possible/Likely	12 (17.4)	12 (4.4)	0.0001	4.5 (1.8–10.9)
**CT Finding (%)**	**Cases (N = 64)**	**Controls (*N*** **= 231)**	**P-Value**	**Odds Ratio** **(95% CI)**
Ground glass opacity	61 (95.3)	154 (66.7)	0.0001	3.3 (1.2–9.1)
Interstitial markings	30 (46.9)	39 (16.8)	0.0001	4.3 (2.2–8.2)
Adenopathy	30 (46.9)	91 (39.2)	0.27	–
Septal thickening	21 (32.8)	69 (29.7)	0.64	–
Consolidation	17 (26.6)	65 (28)	0.82	–
Pleural Effusion	16 (25)	97 (41.8)	0.01	–
Nodular findings	15 (23.4)	104 (45)	0.0001	–
Emphysema/bullae	12 (18.8)	31 (13.4)	0.28	–
Linear-reticular opacities	5 (7.8)	1 (0.4)	0.0001	–
Cavitary lesion	5 (7.8)	7 (3)	0.09	–
Cystic changes	3 (4.7)	8 (3.4)	0.64	–
Mass lesion	1 (1.6)	3 (1.3)	0.87	–
Pulmonary embolism	1 (1.6)	3 (1.3)	0.87	–
Airspace disease	0 (0)	17 (7.3)	0.03	–
Pneumomediastinum	0 (0)	5 (2.2)	0.24	–
Crazy paving	0 (0)	7 (3)	0.16	–
Small airway thickening	0 (0)	5 (2.2)	0.24	–
Pneumothorax	0 (0)	2 (0.9)	0.46	–
Honeycombing	0 (0)	1 (0.4)	0.6	–
Radiologist - PCP Possible	21 (32.8)	50 (21.6)	0.06	2.0 (1.0–4.1)
Radiologist - PCP Likely	16 (25)	8 (3.4)	0.0001	9.3 (3.4–25.3)

Sorted by descending prevalence in PCP. Data is expressed as the number and percentage of total reports in which these findings appeared in. *Ground glass infiltrates or opacities* refers to a nonspecific finding on computed tomography (CT) scans wherein there is partial filling of air spaces in the lungs, as well as interstitial thickening or partial collapse of lung alveoli. May be seen diffusely in the lung; “ground glass” indicating 1–2 mm. *Reticulonodular opacities or linear-reticular opacities* refers to overlaying of reticular shadows (irregular linear opacities) and this can also be seen in the presence of pulmonary nodules. *Crazy paving* refers to the appearance of ground-glass opacities with superimposed inter and intralobular septal thickening and intralobular septal thickening. *Honeycombing* is seen with widespread fibrosis; small cystic spaces are observed with irregularly thickened walls made up of fibrous tissue

**Table 2:** Probability of PJP based on CT findings

Finding	Points	Total Score	Probability
**Including Radiologist Impression**			
Ground glass opacities	5.5	0	< 5%
Increased interstitial markings	6.5	3	5–10%
Radiologist Impression		5.5	10–15%
PJP Unlikely or Not Mentioned	0	6.5	15–20%
PJP possible	3	8.5	20–25%
PJP likely	10	9.5	25–30%
		12	35–40%
		13	40–45%
		15	50–55%
		15.5	55–60%
		16.5	60–65%
		22	85–90%
**Without Radiologist Impression**			
Absence of nodular disease	5	≤5	< 5%
Absence of pleural effusion	5	9	5–10%
Ground glass opacities	9	10	5–10%
Increased interstitial markings	10	14	10–15%
		15	15–20%
		19	25–30%
		20	25–30%
		24	40–45%
		29	60–65%

To estimate probability, add up the number of points from the two left columns and then look up total score in the third and fourth columns. Provided with or without overall radiologist impression. Probability is based on the pre-test probability in this cohort (11.5%)

## Discussion

We found that the chest x-ray was poor at differentiating PJP from other diagnoses (c-statistic 0.64, poor) within a cohort of patients with a clinical suspicion of PJP, whereas a CT scan could remain helpful (c-statistic 0.75; fair). Neither imaging modality performed well enough to preclude further testing; however, separating patients into lower and higher risk for PJP based on imaging could allow for better use of non-invasive testing (e.g. beta-D-glucan) in patients at low risk of disease and expedited invasive testing in those at higher risk.

While there have been other studies which describe the findings of CT thorax in patients with PJP, only a few small studies have compared them with a clinically relevant control group. Richards et al. found that ground glass opacities on CT were predictive of *P. jirovecii* diagnosed on bronchoscopy in the 4 cases of PJP out of 13 HIV positive patients tested [9]. Similarly, Hidalgo et al. evaluated 30 patients with HIV and presumed PJP and found that diffuse or upper lobe predominant ground glass opacifications were present in all 24 patients with PJP versus 2 of the 6 without [10]. Finally, Gruden et al. evaluated 33 patients with HIV who underwent bronchoscopy for PJP and found that nodular or patchy ground glass opacities were present in all 6 patients with PJP and 5/27 without [11]. The major strengths of our study are our inclusion of a mixed cohort of HIV positive and negative patients and our use of a comparator group who were clinically suspected of having PJP. Consequently, our cohort is a fairer representation of the population that clinicians will encounter when they clinically suspect PJP.

Nonetheless, our study has several limitations. First, this was a single centre study and we are an academic referral hospital. We see PJP frequently and suspected cases are reviewed by dedicated chest radiologists who may have more experience with PJP than other centres. To account for this, we also constructed a model for CT scan without the radiologist’s final impression. Furthermore, we do not use a structured means of recording the clinical details on our radiology requests. Hence, it is possible that requisitions which explicitly included the diagnosis of PJP or a more detailed clinical histories may have biased our radiologists towards reporting features positively associated with the diagnosis. We also cannot say for certain that all radiographic features included in Table 1 were systematically assessed by our radiologists as our institution does not use structured reporting templates. Secondly, while we present more cases than all previous studies, our sample size remains limited. Thirdly, patients with a false negative BAL may have been included in the control group and this would bias all estimates towards the null. Fourth, there could be misclassification bias due to false negative bronchoscopy results representing an imperfect gold standard; however, the BAL was considered the best possible reference. Finally, we were only able to abstract radiographic and not clinical data. A more refined clinical prediction model that includes clinical, radiographic, and non-invasive laboratory data would be expected to further improve the ability to estimate probability of PJP. Despite these limitations, we believe our results can help clinicians and be validated in other cohorts.

## Conclusion

In patients for whom there is clinical suspicion of PJP, the use of CT scan can help estimate the probability of PJP. For low risk patients, non-invasive laboratory testing could be appropriately performed and interpreted whereas high risk patients could be considered for empiric therapy and more specific or invasive testing.

## Data Availability

The datasets generated and/or analysed during the current study are not publicly available due to Québec law but are available from the corresponding author on reasonable request and subject to a legal materials transfer agreement.

## Abbreviations

**BAL**: Bronchoalveolar lavage
**CT**: Computed tomography
**HIV**: Human immunodeficiency virus
**PJP**: *Pneumocystis jirovecii* pneumonia
